# Dietary Bentonite Supplementation Improves Growth Performance of Juvenile *Nile tilapia* Under Sub-Acute Aflatoxin B_1_ Exposure

**DOI:** 10.3390/jox16040145

**Published:** 2026-08-03

**Authors:** Lane Pineda, May Moh Moh Khin, Saravanan Subramanian, H.V.L.N. Swamy, Hoang Nhu Phuc, Tran Huu Loc

**Affiliations:** 1 Trouw Nutrition, Stationstraat 77, 3811 MH Amersfoort, The Netherlands; saravanan.subramanian@selko.com (S.S.);; 2 ShrimpVet Laboratory, 307 1A Highway, Tam Gian Housing Estate, Quarter 6, Linh Trung Ward, Thu Duc City, Ho Chi Minh City 720371, Vietnam; may.shrimpvet@gmail.com (M.M.M.K.); phuchoangshrimpvet@gmail.com (H.N.P.); thuuloc@arizona.edu (T.H.L.); 3 Department of Aquatic Animal Pathology, Faculty of Fisheries, Nong Lam University, Quarter 33, Linh Xuan Ward, Ho Chi Minh City 700000, Vietnam

**Keywords:** aflatoxin B_1_, bentonite, mycotoxin binder, *Nile tilapia*, smectite

## Abstract

Previous studies evaluating bentonite in *Nile tilapia* have largely employed high-dose AFB_1_ challenge models (≥2 ppm in the diet), whereas information on its efficacy under sub-acute contamination conditions is limited. This study evaluated the effects of a bentonite-based binder on growth performance and selected gross health-related indices in juvenile *Nile tilapia* exposed to 250 ppb dietary AFB_1_. In a 42-day feeding trial, 320 fish were randomly assigned to four treatments: a negative control (NC; no AFB_1_ or binder), a positive control (PC; 250 ppb AFB_1_), and two AFB_1_-contaminated diets supplemented with bentonite at 1 kg/ton (TMX-1) or 2 kg/ton (TMX-2). Dietary AFB_1_ exposure to 250 ppb impaired growth and feed intake and increased body-weight variability compared with the NC group (*p* < 0.01). Bentonite supplementation elicited inclusion-level-dependent responses. Fish fed TMX-2 had higher final body weight (+7.0%) and average daily gain (+7.9%) than those fed the PC diet (*p* < 0.01), whereas TMX-1 performed similarly to the PC group. Feed conversion ratio differed among treatments, with TMX-2 lower than TMX-1 (*p* = 0.02) but not significantly different from the NC or PC groups. Mortality, condition factor, body-weight variation and organosomatic indices did not differ among AFB_1_-challenged treatments. These findings indicate that dietary bentonite supplementation, particularly at 2 kg/ton, partially alleviated adverse responses associated with sub-acute AFB_1_ exposure, primarily through improvements in final body weight and average daily gain, in juvenile *Nile tilapia* under the conditions of the present study.

## 1. Introduction

Aflatoxins are toxic secondary metabolites produced by *Aspergillus flavus* and *Aspergillus parasiticus* and remain a persistent concern for aquafeed safety [1]. Among these compounds, aflatoxin B_1_ (AFB_1_) is the most prevalent and toxic, with well-documented hepatotoxic, immunotoxic, and carcinogenic effects across aquatic species [2,3,4,5,6,7]. In *Nile tilapia* (*Oreochromis niloticus*), AFB_1_ has been associated with impaired growth, hepatic lesions, oxidative stress, and altered physiological responses [2,5,8]. It may also compromise immune competence and disease resistance, thereby increasing health risks under production conditions [6].

Mycotoxin binders are widely used to reduce AFB_1_ bioavailability in animal production systems, and their application in aquaculture has increased with the growing inclusion of plant-based feed ingredients [9,10,11,12]. Greater reliance on soybean and oilseed meals has reduced pressure on marine resources but has also increased the risk of fungal contamination and mycotoxin formation during production, harvest, and storage [6,7]. Although improved ingredient screening and storage practices are essential, complete prevention remains challenging. In-feed binders are therefore often used to adsorb aflatoxins in the gastrointestinal tract and limit their absorption [12,13,14]. Smectite clays, including bentonite and montmorillonite, are particularly effective because their expandable interlayer structure and high surface area allow them to bind aflatoxins through hydrogen bonding and van der Waals interactions, forming stable complexes across a wide range of gastrointestinal pH conditions and thereby reducing systemic toxin exposure [12,15,16].

Aquaculture studies have shown that bentonite can mitigate the adverse effects of AFB_1_ in tilapia. Dietary calcium bentonite (0.5–1.0%) improved growth performance, feed efficiency, and tissue integrity in *Nile tilapia* challenged with 2–4 ppm AFB_1_ [17], while sodium bentonite enhanced immune responses and reduced hepatic damage at AFB_1_ concentrations of 10–40 ppm [2,18]. Although these studies demonstrate the protective potential of bentonite, they were conducted under relatively high AFB_1_ challenge levels that exceed the concentrations typically reported in commercial aquafeeds. Survey data indicate that AFB_1_ contamination in fish feeds is generally within the range of 10–50 ppb, although higher concentrations may occur because of poor ingredient quality, fungal growth, or inadequate storage [3,10,19]. Consequently, there is limited information on the efficacy of bentonite under more moderate contamination levels that may better represent practical production conditions. Evaluating bentonite supplementation under elevated but sub-acute AFB_1_ exposure is therefore important for developing evidence-based recommendations for its use in aquafeeds.

The objective of this study was to evaluate the effects of dietary bentonite supplementation at two inclusion levels (1 or 2 kg/ton of feed) on growth performance and selected gross health-related indices, including survival, condition factor, hepatosomatic index, and viscerosomatic index, in *Nile tilapia* exposed to a 250-ppb dietary AFB_1_. This concentration was selected as a controlled sub-acute, non-lethal challenge level that is substantially lower than commonly used high-dose models (≥2 ppm), but sufficient to induce measurable biological responses during a 42-day feeding period. The study was designed to determine whether bentonite supplementation could mitigate adverse responses associated with sub-acute AFB_1_ exposure, particularly growth impairment, under an elevated-risk contamination scenario relevant to aquaculture feed production.

## 2. Materials and Methods

### 2.1. Ethical Approval

All experimental procedures, animal handling, and research activities were carried out in compliance with the Law on Animal Health (No. 79/2015/QH13) and the Law on Fisheries (No. 18/2017/QH14) of Vietnam.

### 2.2. Experimental Fish, Treatments and Diet Preparation

Healthy juvenile *Nile tilapia* (*Oreochromis niloticus*; initial weight: 1.0 ± 0.1 g at procurement) were obtained from a certified hatchery in Dong Thap Province, Vietnam. Before transport, fish were screened for *Streptococcus agalactiae*, *Streptococcus iniae*, and *Aeromonas* spp. using polymerase chain reaction (PCR). Fish were then transferred to the experimental facility and reared for two months in 3000 L tanks equipped with airlift biofilters under controlled conditions. Temperature, dissolved oxygen (DO), and pH were maintained within ranges suitable for *Nile tilapia* under a 12 h light:12 h dark photoperiod.

After the two-month pre-trial rearing period, 320 fish (mean body weight ≈ 11.3 g) were assigned to four dietary treatments according to a randomized block design and stocked into 250 L tanks (20 fish per tank; four replicate tanks per treatment) for a 42-day feeding trial following a 2-day acclimation period. The treatments were: (1) a negative control (NC) diet without AFB_1_ or binder; (2) a positive control (PC) diet containing 250 ppb AFB_1_; (3) the PC diet supplemented with TOXO-MX at 1 kg/ton (TMX-1); and (4) the PC diet supplemented with TOXO-MX at 2 kg/ton (TMX-2). TOXO-MX (Trouw Nutrition, Amersfoort, The Netherlands) is a bentonite-based product with a high content of smectite minerals, designed to support growth performance and health under mycotoxin exposure conditions.

### 2.3. Diets

Four experimental diets were formulated according to the following objectives. First, a common basal diet was formulated to meet the nutritional requirements of tilapia [20] using fish meal, soybean meal, rice bran, and wheat flour (Table 1). Second, to establish an aflatoxin challenge, purified AFB_1_ was added at 250 ppb to the basal diet, resulting in three challenged diets: the PC, TMX-1, and TMX-2. Third, to evaluate the efficacy of a bentonite-based binder (TOXO-MX), the product was included at 0.1% and 0.2% in TMX-1 and TMX-2, respectively. Thus, all treatment groups received the same basal diet with identical ingredient composition and nutrient content, with the only differences among treatments being the inclusion of AFB_1_ and/or TOXO-MX according to the experimental design.

The basal diet was prepared in mash form from a single batch of ingredients, after which the experimental diets were produced using a manual pelletizer according to the treatment design. Purified AFB1 (Pribolab Pte. Ltd., Singapore) was premixed with wheat flour for 10 min before incorporation into the designated diets. TOXO-MX was then added to the appropriate diets, homogenized, pelleted into 1.3-mm sinking pellets, and dried at 40 °C for 5–6 h. After drying, a Nustic binder (Bayer AG Crop Science Division, Monheim am Rhein, Germany) was applied, followed by re-drying to ensure uniform coating and pellet integrity. Diets were stored at 4 °C until use.

Representative samples of the basal diet were collected for proximate composition and background aflatoxin analysis before supplementation. Following AFB1 addition, samples of the challenged diet were analyzed to confirm the target AFB1 concentration (Table 2). Dietary AFB1 concentrations were quantified using high-performance liquid chromatography with fluorescence detection (HPLC-FLD; CH038.2021 method) at a commercial laboratory in Vietnam.

### 2.4. Management

Fish were offered feed to apparent satiation, divided into four meals per day at 08:00, 11:00, 14:00, and 17:00. The feeding rate was adjusted daily based on the estimated biomass in each tank and observed feeding behavior. Biomass was estimated by periodically weighing a representative sample of fish from each tank and extrapolating to the total population. Feed consumption in each tank was recorded daily. After each meal, uneaten feed was removed using a fine-mesh net to collect floating pellets, followed by siphoning to recover any sinking or fragmented feed. The collected material was dried at 60 °C for 5–6 h and weighed to determine the amount of uneaten feed.

### 2.5. Water Quality Monitoring

Water quality was monitored and maintained throughout the trial. Temperature, DO, and pH were measured daily, whereas total ammonia nitrogen (TAN) and nitrite (NO_2_^−^) were measured twice weekly. Across treatments, water temperature ranged from 27.90 ± 0.12 to 27.96 ± 0.11 °C, DO from 6.44 ± 0.06 to 6.51 ± 0.07 mg L^−1^, pH from 7.94 ± 0.09 to 7.96 ± 0.09, TAN from 0.89 ± 0.45 to 0.93 ± 0.55 mg L^−1^, and nitrite from 1.71 ± 1.07 to 1.93 ± 0.92 mg L^−1^ (Table 3). To prevent nutrient accumulation and maintain stable water quality, 30% of the water in the 250 L static tanks was replaced every five days.

### 2.6. Measurements

Fish were monitored for growth performance, survival, condition factor, and organosomatic indices throughout the trial. On day 42, individual body weight was recorded. Average daily feed intake (ADFI) was calculated as the total dry matter consumed per tank per day divided by the number of fish to obtain average daily dry matter intake per fish. Growth indices, including average daily gain (ADG), specific growth rate (SGR), and feed conversion ratio (FCR), were calculated. The coefficient of variation (CV) of body weight at the end of the study was calculated from the individual final body weights recorded within each replicate tank. Mortality was recorded daily, and survival was calculated at the end of the experiment. In addition, three fish per replicate (12 fish per treatment) were randomly selected on day 42 for organ sampling. Before biometric measurements and tissue collection, fish were sedated by immersion in ice-chilled water (approximately 5–10 °C) until cessation of swimming activity and loss of the righting reflex, consistent with a surgical plane of sedation. Fish were then euthanized using the same ice-water immersion method and maintained in the ice-water bath until opercular and body movements had ceased, followed by an additional period to confirm death before sample collection. Fish were blotted dry with sterile gauze to minimize contamination. The liver and viscera were carefully dissected using clean surgical scissors, with a consistent technique applied across all specimens. Organs were weighed immediately after removal to calculate the hepatosomatic index (HSI) and viscerosomatic index (VSI).

Growth performance, feed utilization, and somatic indices were calculated as follows:

Survival rate (%) = Final number of fish/Initial number of fish × 100,(1)

ADG (g day^−1^) = (Final weight − Initial weight)/Culture period (days), (2)

SGR (% day^−1^) = [ln (Final mean weight) − ln (Initial mean weight)]/Culture period (days) × 100, (3)

FCR = Total feed intake (g DM)/Fish biomass gain (g),(4)

CV of body weight (%) = (Standard deviation/Mean body weight) × 100,(5)

Condition factor = Body weight (g)/Body length^3^ (cm^3^) × 100. (6)

HSI (%) = Liver weight (g)/Body weight (g) × 100,(7)

VSI (%) = Viscera weight (g)/Body weight (g) × 100,(8)

### 2.7. Statistical Analysis

All data were assessed for normality using the Shapiro–Wilk test and for homogeneity of variance using Levene’s test. Model assumptions were met for all response variables; therefore, no data transformations were required. Data were analyzed using the MIXED procedure in SAS Studio 3.8/SAS 9.4 (SAS Institute Inc., Cary, NC, USA), with treatment included as a fixed effect and block included as a random effect. When a significant treatment effect was detected, treatment means were separated using Tukey–Kramer’s test. Statistical significance was declared at *p* ≤ 0.05. Exact *p*-values are reported to two decimal places, except values below 0.01, which are reported as *p* < 0.01. Values between 0.05 and 0.10, when present, were interpreted descriptively and were not considered statistically significant.

## 3. Results

### 3.1. Water Quality

Water quality parameters were maintained within ranges suitable for *Nile tilapia* throughout the experimental period (Table 3), ensuring that environmental variables did not confound treatment effects.

### 3.2. Growth Performance

Dietary AFB_1_ at 250 ppb impaired growth performance, whereas survival remained 100% across all treatments (Table 4). Relative to the NC group, fish in the PC group showed lower final body weight (−13.6%), ADG (−15.8%), SGR (−7.3%), and ADFI (−11.2%) (*p* < 0.01), while FCR did not differ between the NC and PC groups.

**Figure 1:**
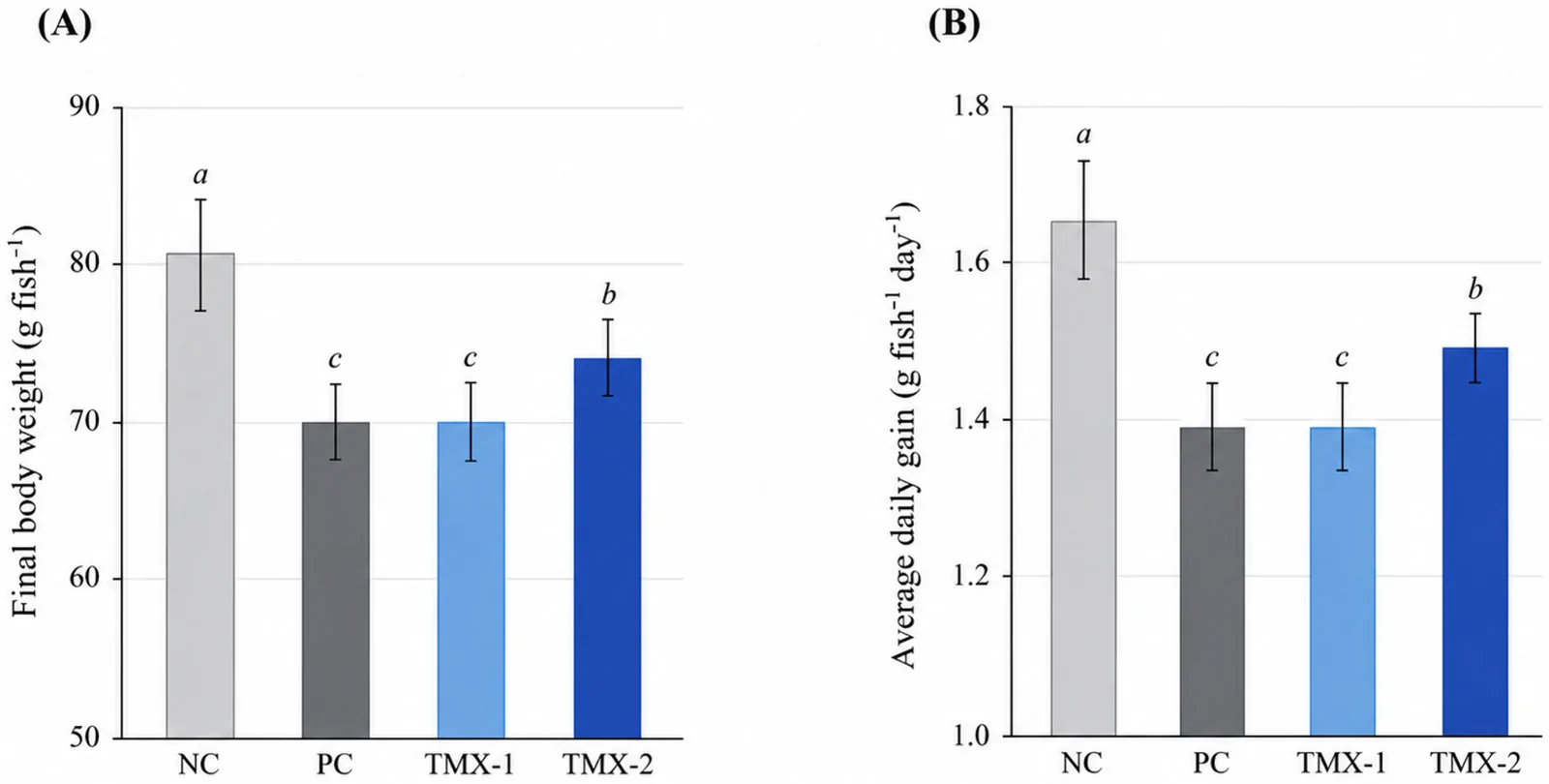
Effects of dietary bentonite supplementation on final body weight (**A**) and average daily gain (**B**) in juvenile *Nile tilapia* under sub-acute aflatoxin B_1_ exposure. Values are presented as mean ± SD of replicate tanks (n = 4). Different letters above bars indicate significant differences among treatments based on Tukey–Kramer’s test (*p* ≤ 0.05). NC = negative control; PC = positive control diet containing 250 ppb AFB_1_; TMX-1 = PC diet supplemented with TOXO-MX at 1 kg/ton; TMX-2 = PC diet supplemented with TOXO-MX at 2 kg/ton.

TMX-2 supplementation increased final body weight (+7.0%) and ADG (+7.9%) compared with both the PC and TMX-1 groups (*p* < 0.01; Figure 1). SGR in the TMX-2 group was intermediate between the NC and PC groups and did not differ significantly from either group. FCR differed among treatments (*p* = 0.02); however, TMX-2 did not differ from either the NC or PC group. In contrast, TMX-1 did not differ from the PC group for any measured growth performance parameter.

Supplementation with TMX-1 or TMX-2 reduced the CV of final body weight relative to the PC group; however, these differences were not statistically significant. ADFI and condition factor remained comparable among the AFB_1_-challenged treatments (Table 4).

### 3.3. Hepatosomatic and Viscerosomatic Indices

No significant differences in HSI or VSI were observed among treatments (*p* > 0.05; Table 5), suggesting that dietary AFB_1_ exposure at 250 ppb and bentonite supplementation did not induce detectable changes in these organosomatic indices during the 42-day experimental period.

## 4. Discussion

This study demonstrates that dietary exposure to 250 ppb AFB_1_ impaired growth performance in juvenile *Nile tilapia*, consistent with previous reports in fish exposed to dietary AFB_1_ [8,21,22] and with aflatoxin-induced metabolic disruption. Following ingestion, AFB_1_ is bioactivated in the liver by cytochrome P450 enzymes to the reactive AFB_1_-8,9-epoxide, which interferes with protein synthesis and disrupts hepatic function [9,23,24,25]. Because the liver is central to amino acid metabolism, lipid transport, and energy homeostasis, this impairment compromises nutrient metabolism and growth [9,22]. Additionally, aflatoxin exposure may induce oxidative stress and intestinal damage, further limiting digestive efficiency and nutrient absorption [5,25]. The observed reduction in feed intake may also reflect altered neuroendocrine regulation associated with hepatic stress and appetite regulation in fish [26,27].

Supplementation with bentonite (TMX) partially alleviated some adverse effects associated with dietary AFB_1_ exposure. At the higher inclusion level (TMX-2), a significant improvement in final body weight was observed compared with both the AFB_1_-challenged control and TMX-1. In contrast, TMX-1 did not differ significantly from the AFB_1_-challenged control for the measured growth performance parameters, suggesting that the efficacy of the binder was dependent on its inclusion level under the applied AFB_1_ challenge conditions. Although AFB_1_ bioavailability was not directly measured in the present study, the improved growth performance and feed efficiency observed in TMX-2 suggest that bentonite may have reduced AFB_1_ bioavailability and toxicity by adsorbing the toxin within the gastrointestinal tract, thereby limiting its absorption and systemic exposure, as reported previously for clay-based binders and bentonite sources [11,12,13,14,15,16,17,18].

Although SGR showed a non-significant trend towards improvement in TMX-2, neither bentonite level significantly affected feed intake or condition factor. These findings suggest that differences in feed intake alone do not explain the observed growth responses; however, the underlying mechanisms remain unclear and warrant further investigation. Although FCR differed between TMX-1 and TMX-2, neither treatment differed significantly from the NC or PC groups, indicating limited evidence that bentonite supplementation improved feed utilization under the conditions of the present study.

These findings align with previous reports demonstrating that clay-based binders may reduce aflatoxin bioavailability and toxicity in aquatic and terrestrial animals [9,11,12,13,14,15,16,17,18,28,29,30]. In *Nile tilapia*, dietary bentonite inclusion has improved growth under aflatoxin challenge [17,18], with similar protective effects reported in poultry and livestock [16,28,29,30]. The absence of changes in feed intake suggests that factors other than feed consumption may have contributed to the observed differences in growth performance.

AFB_1_ exposure also increased body weight variation, as indicated by a higher CV in the challenged groups compared with the NC group (*p* < 0.05). Both bentonite treatments reduced CV relative to the PC group; however, these differences were not statistically significant. This non-significant response may be attributed to limited sample size, short exposure duration, or the inclusion levels tested. Although not statistically significant, the observed improvement in growth uniformity may still be relevant under commercial aquaculture conditions, where size variation affects grading efficiency and harvest management. Further studies with larger sample sizes, different AFB_1_ levels, varying binder inclusion rates, and longer exposure periods are required to clarify the effect of bentonite on growth uniformity under sub-acute exposure.

Survival rates remained at 100% across all treatment groups, indicating that 250 ppb AFB_1_ did not induce acute mortality during the trial. This is consistent with previous studies reporting that tilapia can tolerate dietary aflatoxin exposure without mortality [21]. Deng et al. [21] likewise reported no mortality in fish exposed to higher dietary AFB_1_ levels over extended feeding periods. Although non-lethal, the concentration used in the present study remains relevant, as it exceeds regulatory limits and falls within the range associated with chronic aflatoxicosis [6,31].

No significant differences in HSI or VSI were observed among treatments, suggesting that dietary AFB_1_ exposure at 250 ppb reduced growth performance without corresponding changes in organosomatic indices over the 42-day trial. Previous studies in *Nile tilapia* have reported variable responses in these indices, with some showing significant alterations and others reporting minimal changes under moderate AFB_1_ doses or short-term exposure [32,33]. These inconsistencies are likely attributable to differences in toxin dose, exposure duration, and experimental conditions. While organosomatic indices reflect changes in liver and visceral mass associated with aflatoxicosis, they may lack sensitivity for detecting subclinical or early-stage effects. The stability of HSI and VSI observed in the present study may reflect the moderate exposure level and limited trial duration, which may not have been sufficient to produce measurable changes in these indices. Under such conditions, growth performance parameters, particularly body weight gain and condition factor, may provide more sensitive indicators of sub-acute aflatoxicosis.

Furthermore, the present study evaluated only selected gross health-related indices. Histopathological assessment, oxidative stress biomarkers, immune responses, serum biochemistry, and intestinal integrity measurements were beyond the scope of this study and should be considered in future investigations to further elucidate the protective mechanisms of bentonite.

A limitation of the present study is that diets supplemented with bentonite in the absence of AFB_1_ contamination were not included. Consequently, the independent effects of bentonite on growth performance, feed intake, feed conversion, and selected gross health-related indices under non-challenged conditions could not be determined. Nevertheless, the primary objective of the present study was to evaluate the efficacy of bentonite under sub-acute AFB_1_ challenge conditions, simulating elevated-risk contamination scenarios that may arise due to variability in ingredient quality, fungal proliferation, or storage conditions in aquaculture feed production.

In summary, dietary AFB_1_ at 250 ppb impaired growth performance and increased body weight variability in juvenile *Nile tilapia*. Bentonite supplementation partially alleviated some adverse responses associated with AFB_1_ exposure, with the higher inclusion level (2 kg/ton) improving final body weight and ADG relative to the challenged control. However, definitive evidence for improved feed utilization or reduced aflatoxin bioavailability was not obtained in the present study.

From an applied fish nutrition perspective, bentonite may serve as a practical strategy for managing elevated-risk or sub-acute aflatoxin contamination scenarios in aquafeeds. In the present study, only one AFB_1_ challenge level (250 ppb) was tested; therefore, the results do not allow us to extrapolate the effects of bentonite inclusion under lower levels of AFB_1_ contamination in feed. Further studies are needed to determine the appropriate bentonite inclusion levels under lower AFB_1_ contamination-pressure scenarios.

## 5. Conclusions

Dietary inclusion of 2 kg/ton bentonite partially alleviated the adverse effects of AFB_1_ on growth performance of juvenile *Nile tilapia* under sub-acute exposure. While effects on feed utilization were less consistent, these findings support the use of bentonite as a preventive mitigation strategy within integrated feed quality management programs in aquaculture, particularly in scenarios of elevated aflatoxin risk.

## Figures and Tables

**Table 1:** Ingredient composition and analyzed nutrient composition of the experimental basal diet (as-fed basis).

Ingredient	Inclusion Rate (%)
Wheat flour	16.5
Soybean meal	40.0
Rice bran meal	14.5
Fish meal	20.0
Fish oil	1.0
Soya lecithin	2.0
Premix Fish ^1^	2.0
CMC (pellet binder)	4.0
Analyzed nutrient composition	
Gross energy (kcal/kg) ^2^	3692
Moisture (%)	7.60
Crude protein (%)	38.19
Crude fiber (%)	1.79
Ash (%)	8.19
Crude fat (%)	6.47

^1^ Commercial vitamin–mineral premix with the following composition (per kg of premix): Vitamin A (min) 300,000 IU; Vitamin D_3_ (min) 150,000 IU; Vitamin E (min) 3000 mg; dicalcium phosphate (min–max) 135,000–165,000 mg; iron (FeSO_4_) (min–max) 18,000–22,000 mg; copper (CuSO_4_) (min–max) 9000–11,000 mg; zinc (ZnO) (min–max) 9900–12,100 mg; manganese (MnSO_4_) (min–max) 1800–2200 mg. ^2^ Gross energy was calculated based on ingredient composition. CMC = carboxymethyl cellulose.

**Table 2:** Analyzed aflatoxin B_1_ (AFB_1_) content of the experimental diets.

Treatment	AFB_1_ (ppb)
Negative control (basal diet)	ND ^1^
Positive control (AFB_1_-challenged diet)	251.6

^1^ ND = not detected, below the analytical detection limit. Aflatoxin B_1_ was quantified using HPLC-FLD (CH038.2021 method) at a commercial laboratory in Vietnam. The analysis confirmed no detectable AFB_1_ in the basal diet and verified the intended challenge level in the AFB_1_-contaminated diet. The AFB_1_-challenged basal diet was used to prepare the PC, TMX-1, and TMX-2 diets.

**Table 3:** Water quality parameters recorded during the 42-day feeding trial.

Treatment	Temperature (°C)	DO (mg L^−1^)	pH	TAN (mg L^−1^)	Nitrite (mg L^−1^)
NC	27.96 ± 0.11	6.51 ± 0.07	7.96 ± 0.09	0.93 ± 0.55	1.71 ± 1.07
PC	27.94 ± 0.10	6.46 ± 0.07	7.95 ± 0.09	0.93 ± 0.55	1.79 ± 0.89
TMX-1	27.93 ± 0.09	6.44 ± 0.06	7.96 ± 0.09	0.91 ± 0.57	1.86 ± 1.03
TMX-2	27.90 ± 0.12	6.49 ± 0.09	7.94 ± 0.09	0.89 ± 0.45	1.93 ± 0.92

Values are presented as mean ± SD (n = 4 replicate tanks per treatment). DO = dissolved oxygen; TAN = total ammonia nitrogen.

**Table 4:** Growth performance, coefficient of variation, and condition indices of *Nile tilapia* fed experimental diets for 42 days.

Treatment	Final Body Weight (g fish^−1^)	ADG (g fish^−1^ day^−1^)	ADFI (g fish^−1^ day^−1^)	SGR (% day^−1^)	FCR	CV Final Body Weight (%)	Condition Factor
NC	80.63 ± 2.08 ^a^	1.65 ± 0.06 ^a^	1.79 ± 0.02 ^a^	4.67 ± 0.16 ^a^	1.09 ± 0.04 ^ab^	18.61 ± 1.91 ^b^	2.09 ± 0.05 ^a^
PC	69.68 ± 2.13 ^c^	1.39 ± 0.06 ^c^	1.59 ± 0.01 ^b^	4.33 ± 0.12 ^b^	1.15 ± 0.04 ^ab^	25.41 ± 2.60 ^a^	2.22 ± 0.05 ^b^
TMX-1	69.59 ± 2.01 ^c^	1.39 ± 0.06 ^c^	1.61 ± 0.01 ^b^	4.32 ± 0.18 ^b^	1.16 ± 0.06 ^a^	23.17 ± 3.73 ^ab^	2.32 ± 0.06 ^b^
TMX-2	74.54 ± 3.46 ^b^	1.50 ± 0.08 ^b^	1.62 ± 0.03 ^b^	4.47 ± 0.14 ^ab^	1.07 ± 0.05 ^b^	23.00 ± 2.20 ^ab^	2.32 ± 0.04 ^b^
*p*-value	<0.01	<0.01	<0.01	<0.01	0.02	0.02	<0.01

Values are presented as mean ± SD of replicate tanks (n = 4 tanks per treatment; 20 fish per tank). Different superscript letters within a column indicate significant differences based on Tukey–Kramer’s test (*p* ≤ 0.05). NC = negative control; PC = positive control diet containing 250 ppb AFB_1_; TMX-1 = PC diet supplemented with TOXO-MX at 1 kg/ton; TMX-2 = PC diet supplemented with TOXO-MX at 2 kg/ton; ADG = average daily gain, ADFI = average daily feed intake, SGR = specific growth rate, FCR = feed conversion ratio, CV = coefficient of variation.

**Table 5:** Hepatosomatic index (HSI) and viscerosomatic index (VSI) of *Nile tilapia* at day 42 of the feeding trial.

Treatment	HSI (%)	VSI (%)
NC	2.01 ± 0.52	8.41 ± 1.34
PC	1.73 ± 0.21	8.32 ± 0.83
TMX-1	1.76 ± 0.71	8.48 ± 1.11
TMX-2	1.75 ± 0.45	7.92 ± 0.91
*p*-value	0.58	0.61

Values are presented as mean ± SD (n = 3 fish per replicate; 4 replicates per treatment). No significant treatment effects were detected for HSI (*p* = 0.58) or VSI (*p* = 0.61). NC = negative control; PC = positive control diet containing 250 ppb AFB_1_; TMX-1 = PC diet supplemented with TOXO-MX at 1 kg/ton; TMX-2 = PC diet supplemented with TOXO-MX at 2 kg/ton.

## Data Availability

The data presented in this study are available on request from the corresponding author. The data are not publicly available due to confidentiality restrictions.

## Abbreviations

The following abbreviations are used in this manuscript:

AFB_1_	aflatoxin B_1_
ADFI	average daily feed intake
ADG	average daily gain
CMC	carboxymethyl cellulose
CV	coefficient of variation
DO	dissolved oxygen
FCR	feed conversion ratio
HPLC-FLD	high-performance liquid chromatography with fluorescence detection
HSI	hepatosomatic index
NC	negative control diet without AFB_1_ or binder
ND	not detected
NO_2_^−^	nitrite
PC	positive control diet containing 250 ppb AFB_1_
PCR	polymerase chain reaction
SGR	specific growth rate
TAN	total ammonia nitrogen
TMX	TOXO-MX
TMX-1	PC diet supplemented with TOXO-MX at 1 kg/ton
TMX-2	PC diet supplemented with TOXO-MX at 2 kg/ton
VSI	viscerosomatic index
